# MZF-1/Elk-1 Complex Binds to Protein Kinase Cα Promoter and Is Involved in Hepatocellular Carcinoma

**DOI:** 10.1371/journal.pone.0127420

**Published:** 2015-05-26

**Authors:** Chia-Herng Yue, Chih-Yang Huang, Jen-Hsiang Tsai, Chih-Wei Hsu, Yi-Hsien Hsieh, Ho Lin, Jer-Yuh Liu

**Affiliations:** 1 Department of Life Science, National Chung Hsing University, Taichung, Taiwan; 2 Department of Surgery, Tungs’ Taichung MetroHarbor Hospital, Taichung, Taiwan; 3 Graduate Institute of Chinese Medical Science, School of Chinese Medicine, China Medical University, Taichung, Taiwan; 4 Graduate Institute of Basic Medical Science, China Medical University, Taichung, Taiwan; 5 Department of Health and Nutrition Biotechnology, Asia University, Taichung, Taiwan; 6 Basic Medical Science Education Center, College of Medicine and Health, Fooyin University, Kaohsiung, Taiwan; 7 Department of applied Chemistry, National Chiayi University, Chiayi, Taiwan; 8 Clinical Laboratory, Chung Shan Medical University Hospital, Taichung, Taiwan; 9 Institute of Biochemistry and Biotechnology, Medical College, Chung-Shan Medical University, Taichung, Taiwan; 10 Center for Molecular Medicine, China Medical University Hospital, Taichung, Taiwan; 11 Graduate Institute of Cancer Biology, China Medical University, Taichung, Taiwan; Georgetown University, UNITED STATES

## Abstract

In this study, the molecular mechanism of protein kinase C alpha (PKCα) gene regulation in hepatocellular carcinoma (HCC) involving Ets-like protein-1 (Elk-1) and myeloid zinc finger-1 (MZF-1) was investigated. The luciferase reporter assay results revealed that the presence of both MZF-1 and Elk-1 significantly contributed to the upregulation of PKCα gene transcription activity, and the transcriptional activity decreased when the transfection included a DNA-binding-deficient (∆DBD) gene vector of either MZF-1 or Elk-1 DNA-binding deficiency (MZF-1∆DBD or Elk-1∆DBD), thereby indicating that the enhanced expression of PKCα was caused by the binding of MZF-1 and/or Elk-1 with the PKCα promoter. We investigated MZF-1 and Elk-1 to determine whether they bind to each other. The results of immunoprecipitation (IP), Co-IP, chromatin IP (ChIP), and Re-ChIP analyses indicated that Elk-1 can directly bind to the N-terminal region of MZF-1 and MZF-1 can directly bind to the C-terminal region of Elk-1 to form a complex before attaching to the PKCα promoter. Furthermore, when MZF-1∆DBD or Elk-1∆DBD was added to the cells, PKCα expression decreased, and cell proliferation, migration, invasion, and tumorigenicity also decreased. These findings suggest that PKCα expression in HCC could be stimulated by the formation of MZF-1/Elk-1 complex, which directly binds to the PKCα promoter.

## Introduction

Protein kinase C alpha (PKCα), one of the proteins in the kinase C family, is generally known to serve an important function in tumorigenesis, invasion, and metastasis [1–3]. It has been considered an interesting and challenging target for the development of new therapeutic agents. Although clinical trials have yet to produce a satisfactory result [4], targeting PKCα remains a viable treatment method because of its significant function in tumor cells.

Our recent studies found that two transcription genes, namely, Ets-like protein-1 (Elk-1) and myeloid zinc finger-1 (MZF-1), can regulate PKCα expression [5–7]. Thus, these genes are viable targets for new PKCα inhibition strategies. The Elk-1 protein is capable of activating the c-*fos* promoter with serum response factors (SRFs) and is a target of both extracellular signal-regulated kinase (ERK) and JNK MAP kinase cascades [8]. Elk-1 controls the expression of genes involved in cell cycle progression, differentiation, and apoptosis [9–11] and also serves a central function in cell response to extra-cellular signals. MZF-1, the other protein in this equation, is a transcription factor of the Kruppel family of zinc finger proteins. MZF-1 is preferentially expressed in myeloid progenitor cells [12] and is also involved in the growth, differentiation, and apoptosis of myeloid progenitors [13–15]. Both MZF-1 and Elk-1 gene expressions have been found to be associated with PKCα in liver cancer cells [5–7], thereby suggesting that these transcription factors in PKCα cooperate in influencing carcinogenesis. However, the unfamiliar molecular mechanism remains to be investigated. Thus, this study aims to delineate in greater detail the molecular mechanism underlying PKCα gene regulation by Elk-1 and MZF-1.

This study uses the human hepatocellular carcinoma (HCC) model to demonstrate that MZF-1 and Elk-1 directly bind to the PKCα promoter and modulate PKCα performance through the interactive cooperation between the two transcription factors. The mechanism of a possible formation of a complex (MZF-1 and Elk-1), which binds to the PKCα promoter and enhances PKCα expression and cell growth and invasion, is discussed.

## Materials and Methods

### Materials

Anti-PKCα, δ, ε, and ι purchases were made from BD Biosciences (San Jose, CA); anti-FLAG and anti-Myc from Sigma (St. Louis, MO); and anti-Elk-1, anti-MZF-1, and α-tubulin polyclonal antibodies from Santa Cruz Biotechnology, Inc. (Santa Cruz, CA). Horseradish peroxidase-labeled anti-mouse and anti-rabbit secondary antibodies were obtained from Promega (Madison, WI). Antisense Elk-1 and MZF-1 oligonucleotide were provided by MDBio, Inc. (Taipei, Taiwan). Luciferase reagent and lysis buffer were obtained from Promega Corp. (Madison, WI).

### Cell culture

HA22T/VGH (BCRC No. 60168), Huh-7, Hep3B (BCRC No. 60434), and HepG2 (BCRC No. 60025) were purchased from the Bioresources Collection and Research Center of the Food Industry Research and Development Institute (Hsinchu, Taiwan), whereas SK-Hep-1 was from the American Type Culture Collection (Rockville, MD). The poorly differentiated HA22T/VGH and SK-Hep-1 cell lines and the well-differentiated Huh-7, Hep3B, and HepG2 cell lines^5^ were cultured in Dulbecco's modified Eagle's medium (DMEM) supplemented with 10% fetal calf serum (FCS) (Hyclone, Logan, UT), 2 mM L-glutamine, penicillin (100 units/mL), and streptomycin (100 μg/mL) and then grown at 37°C in 5% CO_2_.

### Construct plasmid

The expression vectors described below were delivered by the cytomegalovirus (CMV) promoter-basic contained in the pcDNA3 vector (Invitrogen, Carlsbad, CA). All open reading frames of human MZF-1 and Elk-1 genes were obtained from the SK-Hep-1 cells by RT-PCR. pcDNA-Elk-1 (GenBank Accession No. AB016193 101–1384 bp) using the primer pairs 5’-CGGGATCCATGGACCCATCTGTGACG-3’, followed by a BamHI site and 5’-CCCAAGCTTTGGCTTCTGGGGCCCTGG-3’ followed by a HindIII site and pcDNA-MZF-1 (GenBank Accession No. AF161886 10781–12235 bp) were amplified by PCR using the primer pairs 5’-GGAATTCCAATGAATGGTCCCCTTGTG-3’ followed by a EcoRI site and 5’-GGGGTACCCTCGGCGCTGTGGACGCG-3’ a KpnI site. PCR amplifications were performed with Super-Therm DNA polymerase (Promega) employed 35 cycles with steps at 94°C for 30 s, 52°C for 30 s, and 72°C for 150 s. The PCR products were isolated and cloned into the pcDNA 3.1/Myc-His vector (Invitrogen). Sequence fidelity of both Elk-1 and MZF-1 was confirmed using DNA sequence analysis (data not shown)

The vector containing FLAG-MZF-1 (1–72 aa) was constructed by expressing MZF-1 (1–72 aa; encoding nucleotides 10,781–10,996 bp) in a pFLAG-CMV vector. Fragment (1–72 aa) of DNA-binding deficient MZF-1 (MZF-1∆DBD) was amplified by PCR from pcDNA-MZF-1. Primer pairs are 5’-GGAATTCAATGAATGGTCCCCTTGTG-3’ and 5’- CCGCTCGAGCCCATCCTCGTCCGTGGGGTC -3’ with EcoRI and XbaI sites, respectively. The PCR products were isolated and cloned into the pcDNA 3.1/Myc-His vector (Invitrogen).

The vector containing c-Myc-Elk-1 (87–428 aa) was constructed by expressing Elk-1 (86–428 aa; encoding nucleotides 359–1384 bp) in a pcDNA 3.1/*Myc*-His (Invitrogen, Carlsbad, CA) vector. Fragment (86–428 aa) of DNA-binding deficient Elk-1 (Elk-1∆DBD) was amplified by PCR from pcDNA-Elk-1. Primer pairs are 5’-GGAATTCCATCCTACCCTGAGGTCGCT-3’ and 5’-CCCAAGCTTTGGCTTCTGGGGCCCTGG-3’ with EcoRI and HindIII sites, respectively. The PCR products were isolated and cloned into the pcDNA 3.1/Myc-His vector (Invitrogen).

### Transient transfection

Transfections were performed using Lipofectamine 2000. Cells seeded in a 60 mm dish were cultured in DMEM supplemented with 10% FCS at 37°C for 24 h. After incubation, the cells were rinsed with serum-free MEM before adding 1 mL MEM containing 10 μg/mL Lipofectamine 2000 Transfection Reagent (Invitrogen, Carlsbad, CA) and 0.5 μg to 5 μg of the indicated plasmid. The cells were then incubated at 37 C for 6 h before adding 1 mL MEM supplemented with 20% FCS to the medium. After incubation at 37°C for 18 h, the medium was replaced with fresh 10% FCS-DMEM, and the cells were incubated at 37°C for 24 h. The cells were then lysed for Western blot analysis and RT-PCR.

### Luciferase reporter assay

A reporter gene assay was measured using the Dual Luciferase Assay System (Promega) following manufacturer's instructions. The cells were grown in 24-well plates. At 70% confluence, the cultures were transfected using Lipofectamine 2000 for 6 h with 1 μg of one of four reporter plasmids containing designated pGL3-60bp PKCα promoters, which are a 60 bp PKCα promoter sequence 5’-ctcgagcacc gggggtcctg aggatgggga aggggcttcc tgctgcggtg ctgaggaagc-3’ (-660–-600) containing a putative binding site for MZF-1/ Elk-1, a 60 bp PKCα promoter mutMZF-1 sequence 5’-ctcgagcacc gggggtcctg aggattttga aggggcttcc tgctgcggtg ctgaggaagc-3’ containing a putative binding site with GGG (-633–-631) replaced with TTT for mutMZF-1/Elk-1, a 60 bp PKCα promoter mutElk-1 sequence 5’-ctcgagcacc gggggtcctg aggatgggga aggggcttaa ggctgcggtg ctgaggaagc-3’ containing a putative binding site with CCT (-621–-619) replaced with AAG for MZF-1/mutElk-1, and a 60 bp PKCα promoter mutMZF-1/mutElk-1 sequence 5’-ctcgagcacc gggggtcctg aggattttga aggggcttaa ggctgcggtg ctgaggaagc-3’ containing a putative binding site with both changes made for mutMZF-1/ mutElk-1. To maintain the same DNA input in all transfection mixtures, the samples were normalized with empty vectors (pGL3). In addition, to assess transfection efficiency, the pCH110 vector contained a β-galactosidase gene was included in the transfection process. After 48 h from transfection, cells were washed and lysed for the measurement of luciferase activity and β-galactosidase activity. Luminescence was measured over a 20 s interval on a plate luminometer and expressed in arbitrary units. β-galactosidase was measured spectrophotometrically at 420 nm. Luciferase activity was determined and normalized to β-galactosidase activity.

### Western blot analysis

The cultured cells were washed twice with PBS and then lysed with a lysing buffer containing 50 mM Tris/HCl (pH 7.4), 2 mM EDTA, 2 mM EGTA, 150 mM NaCl, 1 mM phenylmethylsulfonyl fluoride (PMSF), 1 mM NaF, 1 mM sodium orthovanadate, 1% (v/v) 2-mercaptoethanol, 1% (v/v) Nonidet P40, and 0.3% sodium deoxycholate. The cell lysates were centrifuged at 12000×g and 4°C for 15 min. The supernatant was collected, and the protein concentration was determined using the Bradford method. Equal amounts of protein extracts (50 μg) were subjected to 12.5% SDS-PAGE and blotted onto a polyvinylidene fluoride membrane (Millipore, Belford, MA). After blocking, the membrane was incubated with the specific anti-PKCα, δ, ε, or ι antibody (1:500), anti-Elk-1 (1:500), anti-MZF-1 (1:500), anti-FLAG (1:4000), anti-Myc (1:4000), or α-tubulin antibody (1:2000). The blots were then incubated with HRP-conjugated anti-mouse or anti-rabbit antibody (1:3000) at room temperature for 2 h. Proteins were detected using the enhanced chemiluminescence detection system (Amersham Pharmacia Biotech, Piscataway, NJ).

### Immunoprecipitation and co-immunoprecipitation analyses

Cells were lysed in RIPA buffer [150 mM NaCl, 5 mM EDTA, 50 mM Hepes (pH 7.5), 0.5% (w/v) sodium deoxycholate, 1% Nonidet P-40 (NP-40), and 10 mM 2-mercaptoethanol] containing 2 mM PMSF, 50 mg/mL of aprotinin A, 25 mg/mL of leupeptin, and 25 mg/mL of pepstatin, for 15 minutes at 4°C. Cell lysates were then cleared by centrifugation at 12000×g for 5 min and kept cold on ice. Aliquots of 500 μL of lysate were incubated with 2 μg of specific antibodies. Immunoprecipitates were resolved by 12.5% SDS-PAGE, and the proteins were transferred to polyvinylidine fluoride membranes (Immobilon-P; Millipore, Bedford, MA). Membranes were incubated in blocking buffer [1% (w/v) BSA, 5% (w/v) non-fat dry milk, and 0.1% (v/v) Tween 20 in TBS] overnight at room temperature. Membranes were subsequently probed with the corresponding antibody in blocking buffer for 16 h: anti-MZF-1 antibody (1:500), anti-Elk-1 antibody (1:500), anti-FLAG antibody (1:2000), or anti-Myc antibody (1:2000). Membranes were washed thrice with TBS buffer for 5 min per wash and incubated with a 1:5000 dilution of HRP-conjugated anti-rabbit IgG or anti-mouse IgG for 1 h at room temperature. After three washes with TBS buffer, antibody-reactive proteins were detected using a chemiluminescence substrate (Pierce, Rockford, IL).

### Chromatin Immunoprecipitation (ChIP) Assay

The ChIP assay was performed following the methodology described by Hsieh et al. [5] The SK-Hep-1 cells were treated with or without indicated sense or antisense ODN (5 μM), and cells were harvested and cross-linked with 1% formaldehyde for 10 min. The reaction was then terminated by the addition of 0.25 M Glycine. The procedures thereafter were performed according to Hsieh et al.

### Re-ChIP of chromatin-bound proteins

The methodology described by Reid et al. [16] was followed. Precipitated complexes were eluted from primary immunoprecipitates, pooled from three or four reactions, and incubated with 30 μL of ChIP elution buffer (50 mM NaHCO_3_, 1% SDS). The samples were shaken for 30 min at room temperature, then spun down and collected as supernatants. The complexes were eluted twice, and both elutions were combined. The pooled elutions were diluted 1:10 in buffer (1% Triton X-100, 5 mM EDTA, 150 mM NaCl, 25mM Tris, pH 8) containing a protease inhibitor mixture (Sigma). Further Re-ChIP of supernatants and analysis of the results were performed as described above for primary ChIP immunoprecipitations.

### Antisense knockout assay

The antisense knockout assay was performed according to Hsieh et al. [5], and the following antisense and sense (as a control) sequences were used: Elk-1 (antisense 5′-CAGCGTCACAGATGGGTCCA T-3′, sense 5′-ATGGACCCATCTGTGACGCT G-3′) and MZF-1 (antisense 5′-TACACAAGG GGACCATTCATTC-3′, sense 5′-GAATGAATG GTCCCCTTGTGTA-3′). The procedures thereafter were performed according to Hsieh et al.

### RNA isolation and RT-PCR analysis

Total RNA was isolated from cell specimens using the guanidinium thiocyanate-phenol method. The extract integrity was assessed by 1.5% agarose gel electrophoresis, and RNA was visualized by ethidium bromide staining. The total amount of RNA was determined spectrophotometrically. RT-PCR assay was performed according to De Petro et al. [17] with slight modifications. An aliquot of total RNA (1 μg) was use for RT. The RT product (2 μL) was diluted with the PCR buffer (50 mM KCl, 10 mM Tris-HCl, and 2 mM MgCl_2_) to a final volume of 50 μL, containing 0.5 μM dNTPs (final concentration, 0.8 mM) and 0.5 unit of Super-Therm Taq DNA polymerase (Southern Cross Biotechnology, Cape Town, South Africa). PCR was performed on a GeneAmp PCR system 2400 (Applied Biosystems, Foster City, CA). The ODN primers used in RT-PCR were as described previously [18]. The PCR products were analyzed by 1.5% agarose gel electrophoresis and direct visualization after SYBR Green I (Cambrex Bio Science Rockland, Inc., Rockland, ME) staining. The agarose gel was scanned and analyzed using the Kodak Scientific 1D Imaging System (Eastman Kodak Company, New Haven, CT).

### Cell proliferation/migration/invasion assay

The cell proliferation, migration, and invasion assays were performed according to Hsieh et al. [19].

### Tumorigenicity Assay in Nude Mice

Female BALB/c nude mice, four weeks to six weeks of age, were purchased from National Health Research Institute (Taipei, Taiwan, ROC) and housed in a dedicated nude mouse facility with micro-isolator caging. All procedures involving laboratory animal use were in accordance with the guidelines of the Instituted Animal Care and Use Committee of China Medical University (IACUC, CMU) for the care and use of laboratory animals and all experimental procedures were approved by IACUC-CMU.

The SK-Hep-1 cells were first treated with 5 μg FLAG or MZF-1∆DBD vector, detached by trypsinisation 48 h later, and then washed in triplicate in serum-free DMEM. 1 × 10^7^ cells in 100 μL volume was injected subcutaneously into the right posterior flank of mice by use of a 1 mL syringe with 24-gauge needle^6^. The mice were randomly assigned to a treatment group: FLAG (5 μg, 0.1 mL/mouse) and MZF-1∆DBD (5 μg, 0.1 mL/mouse). Each group had five mice, and the experiment was repeated twice. The tumor volume was calculated by the formula: 0.5236 × L1(L2)^2^, where L1 is long diameter, and L2 is short diameter. The inhibitory rate of tumor growth was calculated by the formula: (tumor volume_FLAG_—tumor volume_MZF-1∆DBD_ / tumor volume_FLAG_) × 100%.

### Statistical analysis

Data were expressed as mean ± SEM and analyzed by analysis of variance. Student’s t-test was used in two-group comparisons. P<0.05 was considered statistically significant.

## Results

### MZF-1 and Elk-1 directly bind to the PKCα promoter

A previous study has identified that poorly differentiated cell lines contain higher expressions of MZF-1, Elk-1, and PKCα genes. To check if the enhanced level of both Elk-1 and MZF-1 were synergistically responsible for the increase in transcriptional activity of PKCα promoter, a luciferase assay was performed in the same cell-lines. S1A Fig shows that the transcriptional activities in the poorly differentiated HA22T/VGH and SK-Hep-1 cells were higher than those in the well-differentiated HepG2, Hep3B, and Huh-7 cells. The activities were consistent with the PKCα, Elk-1, and MZF-1 results in Western blot, in which the expressions in HA22T/VGH and SK-Hep-1 cells were also higher than in other HCC cell lines.

When the MZF-1 vector was transfected to the Huh-7 cells, both MZF-1 and Elk-1 expressions increased, and the transcriptional activities also proportionally increased (S1B and S1C Fig). S1D Fig confirms that in all five HCC cell lines tested, enhanced Elk-1 or MZF-1 increased transcriptional activity. The enhancement of both Elk-1 and MZF-1 were greater than that of Elk-1 or MZF-1 alone in terms of transcriptional activity. These results confirmed previous findings that PKCα expression is regulated by the two transcription genes[5–7].

**Fig 1:**
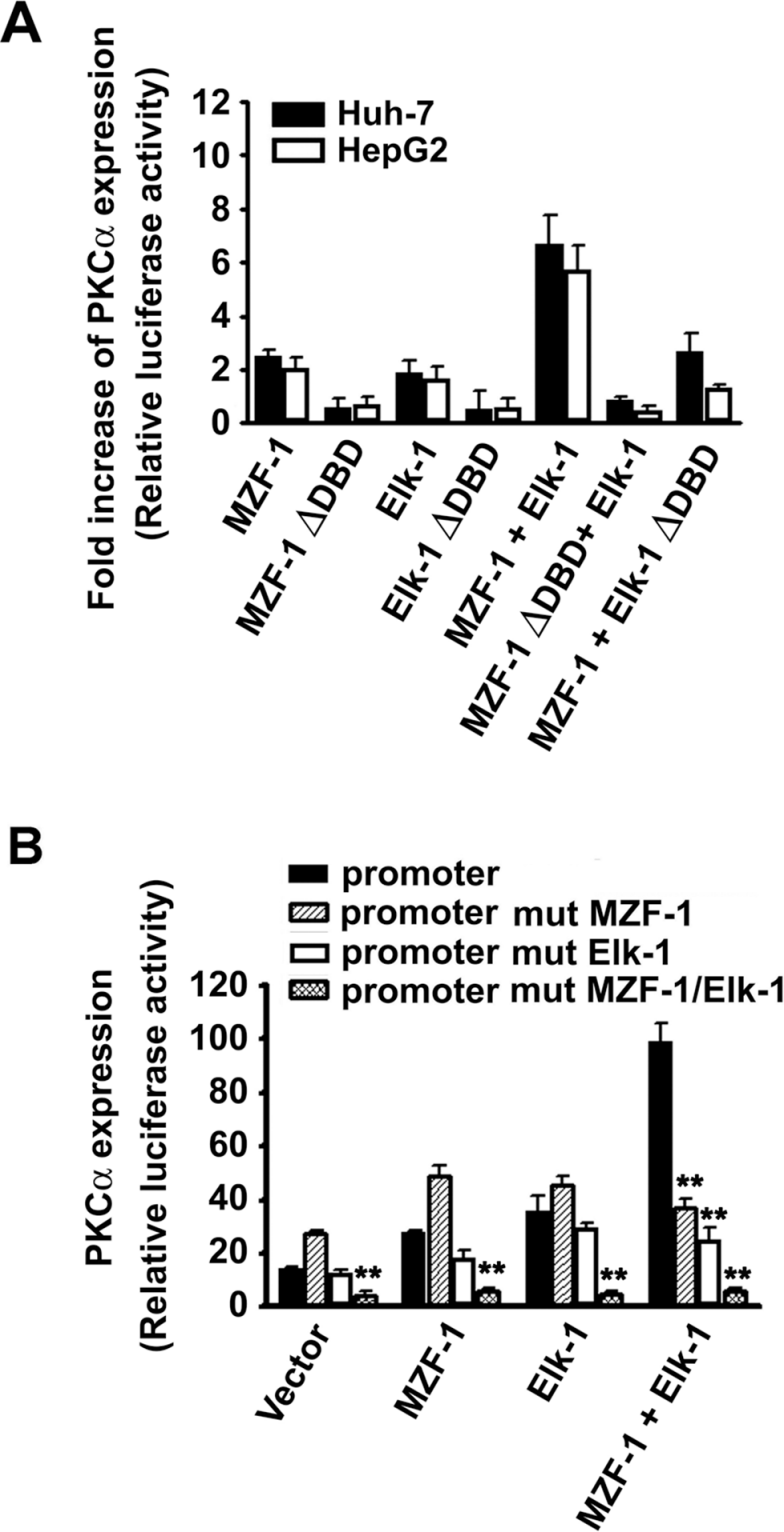
Activation of PKCα expression by combinations of MZF-1 and Elk-1 directly binding to the promoter. (A) Transcriptional activation of PKCα promoter activity by wild-type MZF-1 and Elk-1 and DNA-binding deficient mutants MZF-1 (MZF-1ΔDBD) and Elk-1 (Elk-1ΔDBD) via luciferase assays. MZF-1 and Elk-1 synergistically enhance PKCα transcriptional activity when co-transfected to Huh-7 and HepG2 cells, whereas co-transfection of the DNA-binding domain deletion mutant of MZF-1 (MZF-1ΔDBD)/Elk-1 (Elk-1ΔDBD) failed to enhance the PKCα transcriptional activity. Luciferase activity was normalized to the level of β-galactosidase. Transcriptional activity is expressed as fold induction compared with the level obtained with each reporter vector in the presence of empty pcDNA 3.1/*Myc*-His vectors. The results are the mean ± SE of three independent experiments performed in triplicate. (B) MZF-1 and the Elk-1 binding sites regulate the PKCα promoter activity in Huh-7 cells. Cells were co-transfected with different mutant PKCα promoter luciferase constructs and vectors containing MZF-1 and Elk-1 transcription factors. Luciferase activity was normalized to the level of β-galactosidase. The results are the mean ± SE of three independent experiments performed in triplicate. **, P < 0.01 versus the cells transfected with normal PKCα promoter constructs in each group.

To determine whether transcriptional activity can be regulated by the binding activity of MZF-1 and Elk-1 to PKCα promoters, a mutated form of DNA-binding-deficient (∆DBD) mutant was used. Fig 1A shows that when HepG2 and Huh-7 cells were transfected with either full-length MZF-1 or Elk-1, the transcriptional activity increased compared with their respective control groups (with the empty pcDNA 3.1/*Myc*-His vector). In particular, when both transcriptional factors were used, the activity significantly increased, which is consistent with previous findings^3^. However, when the transfection included a ∆DBD gene vector of either MZF-1 (MZF-1∆DBD) or Elk-1 (Elk-1∆DBD), the transcriptional activity decreased, thereby indicating that the enhanced expression of PKCα is caused by the binding of MZF-1 or/and Elk-1 with the PKCα promoter.

Furthermore, we modified the above mentioned reporter vectors containing Elk-1 and MZF-1 binding sequences in the PKCα promoter by performing minor alterations, as follows: for promoter mut MZF-1, GGG was replaced with TTT in the MZF-1 binding site; for promoter mut Elk-1, CCT was replaced with AAG at the Elk-1 binding site; and for promoter mut MZF-1/Elk-1, both changes were made. The vectors with mutation in the MZF-1 binding site showed a slight increase in transcriptional activity, and the vectors with mutation in the Elk-1 binding site showed a slight decrease in transcriptional activity, whereas the vectors with both MZF-1 and Elk-1 mutations showed a significant decrease in transcriptional activity (Fig 1B). Similar findings were observed in cells with overexpressed MZF-1, overexpressed Elk-1. However, when the cells treated with both overexpressed MZF-1 and Elk-1, the transcriptional activity showed a significant decrease in all mutation types. The mutations in one gene binding site do not completely abolish the transcriptional activity, but the mutations in two gene binding sites nearly abolish the gene expression. Both binding sites function in a gene promoters [20], thereby indicating that the failure of Elk-1 and MZF-1 to bind and a form of mutative PKCα promoter results in lower transcriptional activity.

### MZF-1 and Elk-1 form a complex and bind to the PKCα promoter region

**Fig 2:**
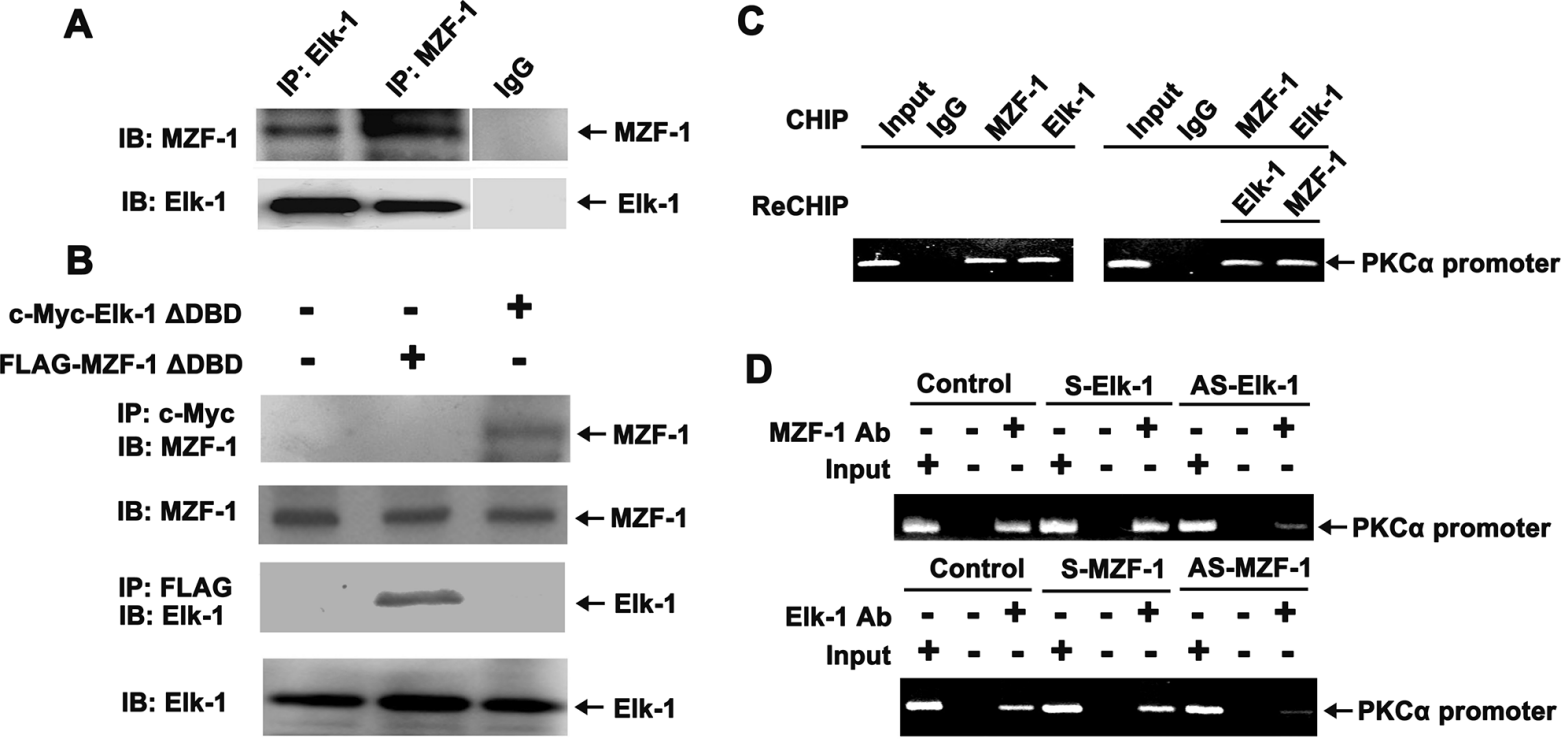
MZF-1 and Elk-1 form a complex at the MZF-1 and Elk-1 sites of the PKCα promoter. (A) Interaction between endogenous MZF-1 and Elk-1 proteins in SK-Hep-1 cells. Protein extracts underwent immunoprecipitation (IP) with anti-MZF-1, anti-Elk-1, or control rabbit IgG, as indicated. Proteins were resolved via SDS-PAGE and then underwent immunoblotting (IB) using anti-MZF-1 or anti-Elk-1 antibodies. (B) N-terminal domain of MZF-1 interacts with Elk-1 at the C-terminal domain of the protein. SK-Hep-1 cells were transfected with expression vectors for FLAG-MZF-1ΔDBD (5 μg) or c-Myc-Elk-1ΔDBD (5 μg), as indicated. Protein extracts underwent IP with anti-Myc and anti-FLAG, as indicated. Proteins were resolved via SDS-PAGE and underwent IB using an anti-MZF-1 or anti-Elk-1 antibody. (C) ChIP and Re-ChIP assays. In the ChIP assay (left), the chromatin was pulled down with IgG, MZF-1, and Elk-1 antibodies, whereas in the Re-ChIP assay (right), the pulled down chromatin interacted with Elk-1 and then with MZF-1 antibodies, after which the sequence was reversed. The PCR products shown are the 210 bp bands in the PAGE result. The input represents the purified chromatin for parallel PCR reaction as a positive control. (D) ChIP assay performed on SK-Hep-1 cells transfected with 5 μM sense or antisense of MZF-1 or with 5 μM sense or antisense of Elk-1. PCR was performed on purified chromatin as control (Input) and on chromatin fragments from the IP with the specific antibodies. The data represent 1 of 3 independent experiments with similar results.

To determine whether MZF-1 and Elk-1 form a complex, a co-IP process was performed with SK-Hep-1 cells. Antibody-bound fractions from the process were subjected to SDS-PAGE and then immunoblotted using the antibody of their counterparts (Fig 2A). The result of IP using the Elk-1 antibody revealed the presence of MZF-1 protein in the immunocomplex, which was in contrast with non-specific IgG IP and was similar to the MZF-1 antibody in Elk-1. Furthermore, when the cells were transfected with c-Myc-Elk-1∆DBD vector, the MZF-1 protein was observed in the immunoprecipitated complex with Myc antibody (Fig 2B). When the cells were transfected with the FLAG-MZF-1∆DBD vector, Elk-1 protein was observed in the immunoprecipitated complex with the FLAG antibody. Therefore, the presence of MZF-1 and Elk-1 in all cells indicated that Elk-1 binds to the N-terminal region of MZF-1, whereas MZF-1 binds to the C-terminal region of Elk-1 during the formation of a complex.

To confirm that endogenous PKCα promoter can be bound by endogenous Elk-1 and MZF-1 complex, chromatin immunoprecipitation (ChIP) and re-chromatin immunoprecipitation (Re-ChIP) assays were performed. The PKCα promoter fragment was amplified from the immunoprecipitated complex with either Elk-1 or MZF-1 antibodies (Fig 2C). In a Re-Chip assay, the amplified products were obtained by first using MZF-1 and then Elk-1 antibody. In another Re-ChIP assay, the order of antibodies used was reversed. In Fig 2D, when the cells were transfected with antisense Elk-1, the quantity of products of the PKCα promoter fragment amplified from the immunoprecipitated complex with MZF-1 antibodies was reduced. No change was observed in the cells transfected with sense Elk-1. Similarly, when the cells were transfected with antisense MZF-1, the quantity of products amplified from the immunoprecipitated complex with Elk-1 antibodies was also reduced, and again, no change was observed in cells transfected with sense MZF-1. These findings indicated the cooperative interaction between Elk-1 and MZF-1, which form a complex and bind to the promoter region of PKCα in SK-Hep-1 cells.

### DBD-truncated-MZF-1 or DBD-truncated-Elk-1 protein interferes with PKCα expression and inhibits cell proliferation/migration/invasion

**Fig 3:**
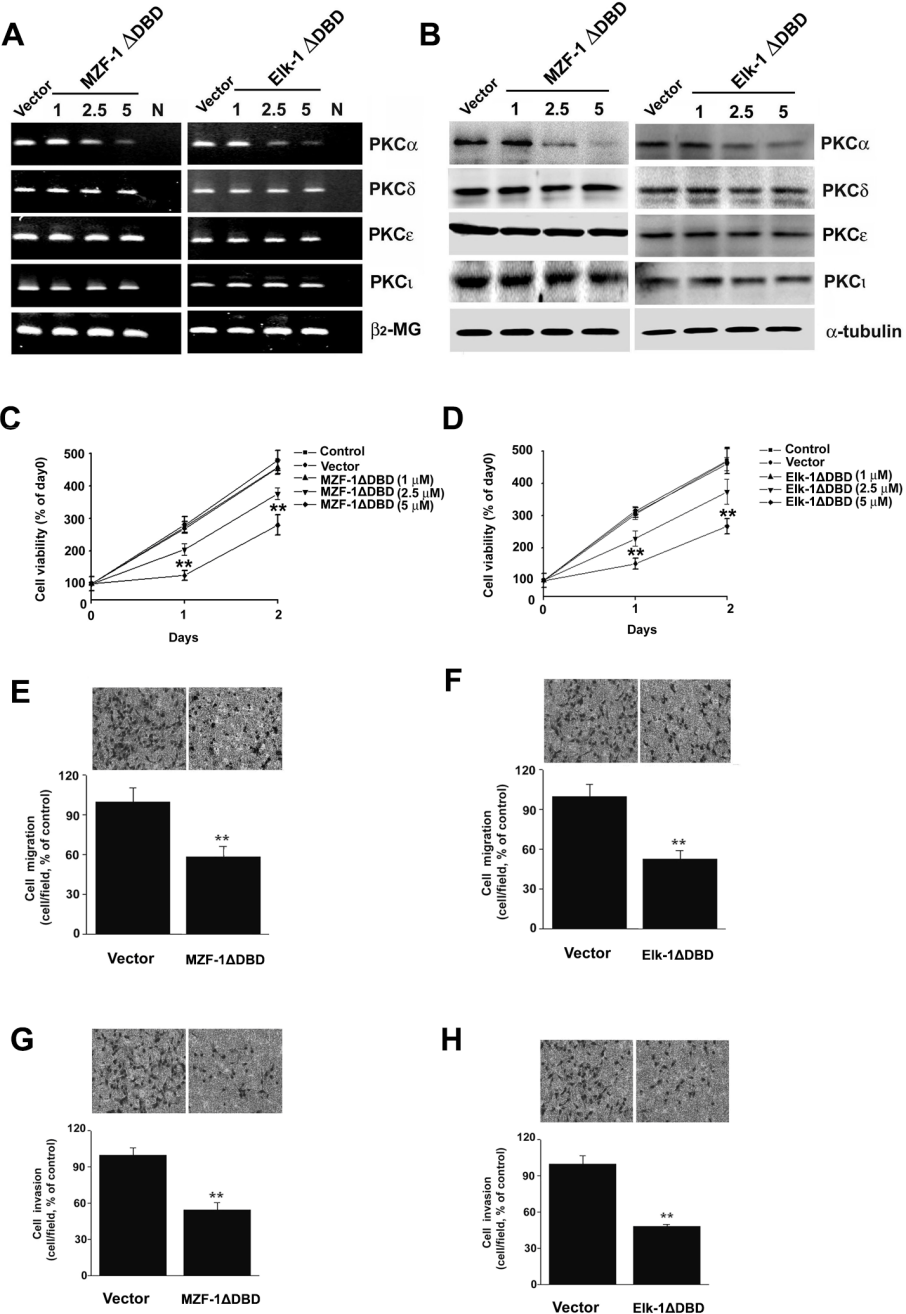
PKCα expression and cell proliferation/migration/invasion inhibition by the truncated-MZF-1 (MZF-1ΔDBD) or-Elk-1 (Elk-1ΔDBD) proteins in SK-Hep-1 cells. The inhibitory effects of transfection with MZF-1ΔDBD and Elk-1ΔDBD constructs from RT-PCR (A) and Western blot analysis (B) assays. SK-Hep-1 cells were transiently transfected with FLAG vector (5 μg), c-Myc vector (5 μg), FLAG-MZF-1ΔDBD (5 μg), or c-Myc-Elk-1ΔDBD (5 μg). (C and D) Effect of MZF-1ΔDBD and Elk-1ΔDBD on cell growth. The SK-Hep-1 cell was transfected with the indicated dose of MZF-1ΔDBD and Elk-1ΔDBD for 1 and 2 d. Untreated cultures were designated as control (Control). Absorbance values obtained from untreated cells on day 0 after subculture were considered 100%. The migration (E and F) and invasion (G and H) assays were performed on cell cultures treated with 5 μg dose of FLAG vector, MZF-1ΔDBD, c-Myc vector, and Elk-1ΔDBD, as described in the Materials and Methods Section. Values are presented as means ± SE of three replicates from two independent experiments. **, P < 0.01 vs. FLAG or c-Myc vector-transfected group.

In the above experiment, the cooperative interaction of Elk-1 and MZF-1 regulated PKCα expression. When the transfection included a ∆DBD gene vector of either MZF-1∆DBD or Elk-1∆DBD, the transcriptional activity decreased compared with their respective control tests (pFLAG-CMV vector or pcDNA-Myc vector). Therefore, we hypothesized that the use of a mutated form of ∆DBD gene can interrupt the endogenous genes when forming a complex and binding to the PKCα promoter, thereby inhibiting gene expression and cell malignancy. When we transfected the MZF-1∆DBD or Elk-1∆DBD gene vector to SK-Hep-1 cells, the PKCα expression decreased, but the other PKC isoenzyme expressions, such as those of δ, ε, or ι, were not influenced (Fig 3A and 3B). Moreover, the MZF-1ΔDBD or Elk-1ΔDBD gene vector showed a dose-dependent reduction in cell proliferation in the same cells (Fig 3C and 3D), and the cell doubling time increased from 103.0% and 104.4% in the control (28.4 h) for 1 μM MZF-1ΔDBD and Elk-1ΔDBD to 148.0% or 147.7% for 2.5 μM. Furthermore, cell proliferation was significantly inhibited by 5 μM MZF-1ΔDBD and Elk-1ΔDBD.

To determine whether MZF-1ΔDBD and Elk-1ΔDBD are inhibited in cell migration/invasion, assays were performed on SK-Hep-1 cells^13^. Both MZF-1ΔDBD and Elk-1ΔDBD inhibited cell migration and invasion in cells by around 50% to 59% compared with the FLAG- or c-Myc-vector-transfected cells (Fig 3E to 3H). This effect further demonstrates the importance of the two genes in cell malignant progression.

### Effect of MZF-1ΔDBD on the tumorigenicity of SK-Hep-1 Cells

**Fig 4:**
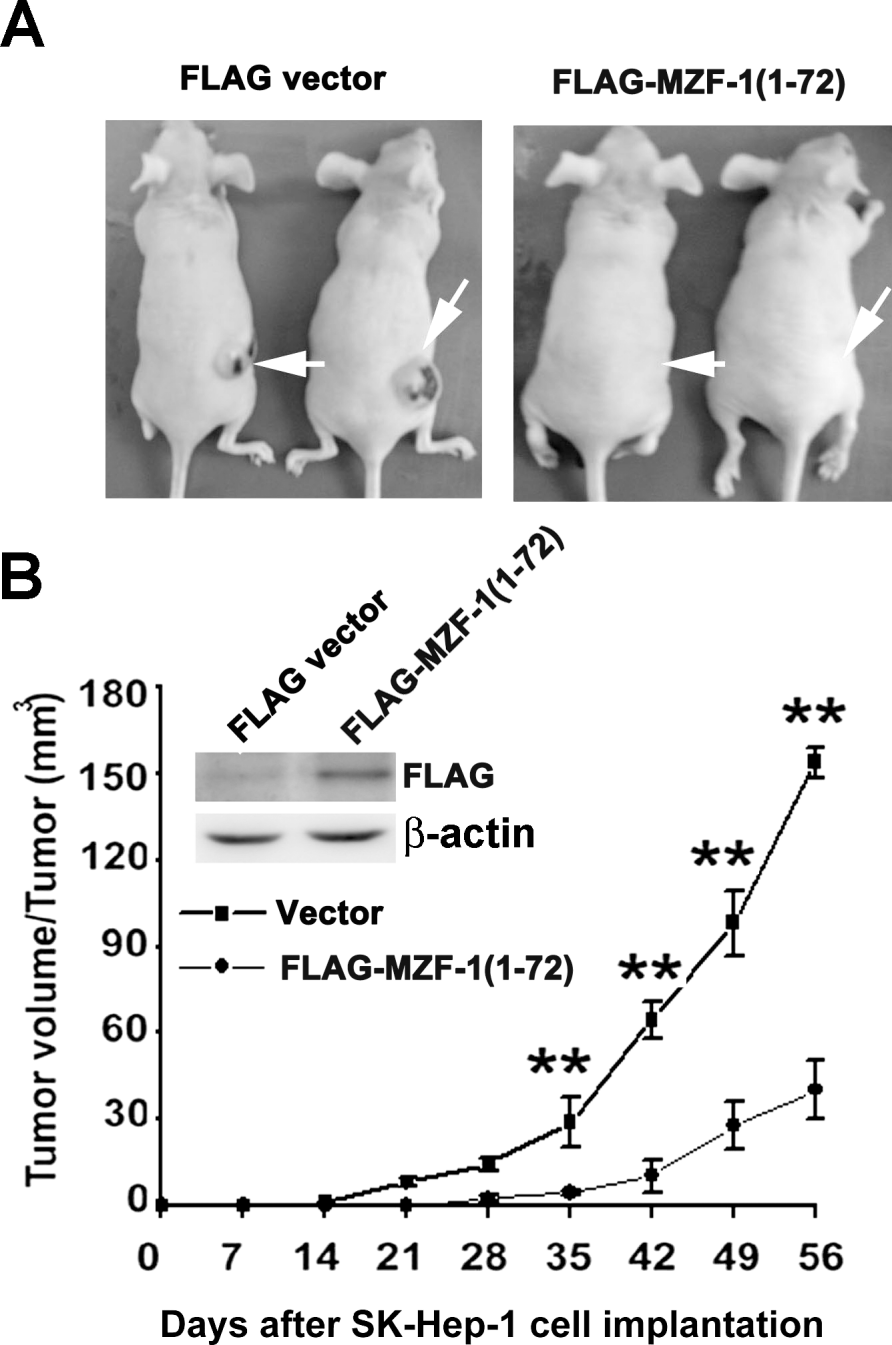
Suppression of tumorigenesis in MZF-1ΔDBD-transfected cells. (A) Tumor growth curve in FLAG (5 μg; ■) or MZF-1ΔDBD (5 μg;●) transfected human SK-Hep-1 xenografts (n = 5). A total of 5 × 10^7^ FLAG- or MZF-1ΔDBD-pretreated cells were injected into the flank region of nude mice. Tumor development was observed in 2 months, and the mice were then sacrificed. Cross-sectional tumor diameters were measured externally, and the approximate tumor volume was calculated as described in the Materials and Methods Section. The results represent the mean ± SE of five developed tumors in the experimental mice. **P < 0.01 vs. FLAG pretreated group. (B) Visible tumor (white arrow) formed by the indicated pretreated cells on day 56. The blots of the anti-FLAG indicated the presence of exogenous MZF-1-(1–72) in cells. β-actin was designed as a housekeeping gene.

Considering that MZF-1ΔDBD specifically inhibited PKCα expression and inhibited cell proliferation/migration/invasiveness, we further examined the effects of MZF-1ΔDBD on tumor formation. The experiment was performed by injecting 5 × 10^7^ FLAG or MZF-1ΔDBD vector-pretreated SK-Hep-1 cells into nude mice, which were then monitored for tumor morphological growth. The FLAG vector-pretreated cells displayed rapid formation and growth of tumors (Fig 4A and 4B). By contrast, the mice injected with MZF-1ΔDBD vector-pretreated cells developed slow-growing tumors. The maximum inhibitory rate of tumor growth was 71.4% ± 9.5% (n = 5), whereas the mean of inhibitory rate of tumor growth from 28 d to 56 d was 70.2% ± 8.6%. The tumor formation time was prolonged from 10 d to 35 d, with a significant difference (P < 0.01) between the MZF-1ΔDBD vector-treated group (28.5 d ± 6.9 d) and the FLAG vector-treated group (16.2 d ± 3.8 d).

## Discussion

The present luciferase reporter data demonstrate that Elk-1 and MZF-1 directly bind to the PKCα promoter and enhance PKCα transcription activity in HCC cells. These findings confirm previous reports that the knockdown of either Elk-1 or MZF-1 can reduce PKCα expression and Elk-1 or MZF-1 over-expression or the overexpression of both can increase PKCα expression in the same cells^3^, thereby suggesting that the cooperation of Elk-1 and MZF-1 may be involved in PKCα gene expression.

Bio-informational analysis results of protein–protein interaction showed that similar functional proteins tend to form a grape-like cluster pattern in cells [21]. Given that the DNA binding sites of Elk-1 and MZF-1 in PKCα promoters are in close proximity to each other [22], we proposed that MZF-1 and Elk-1 may directly interact inside a cluster. Our present data demonstrated that Elk-1 can directly bind to the N-terminal region of MZF-1, whereas MZF-1 can bind to the C-terminal region of Elk-1, and the resulting cluster binds to PKCα promoters. Moreover, when gene knockdown was performed on either MZF-1 or Elk-1, their overall DNA binding capability was compromised. These findings indicated that MZF-1 and Elk-1 do interact cooperatively and affect each other’s binding capability and possibly form a complex.

The formation of a complex bond is a reasonable inference. MZF-1 reportedly has an acidic domain (amino acids 60–72 containing six amino acid of aspartates or glutamates) just upstream of the zinc finger regions [23]. This domain probably functions as a transcriptional activation domain, which is a common feature of various transcriptional factors [24, 25], and is important in the binding with positively charged heparin-binding domains [26]. Heparin-binding sites often contain clusters (XBX, XBBX, and XBBBX) (B = basic residue; X = nonbasic residue) of basic amino acids [arginine (R) and lysine (K)] [27]. In the Elk-1 protein structure, two sequences may contain the following clusters: one (ARSSRNEYMRS; amino acids 146–156) is in the B domain where three clusters can be observed (ARS, SRN, and MRS); and the other (QKGRKPRD; amino acids 311–318) is in the D domain where three clusters are observed (QKG, GRKP, and PRD). The B domain enables Elk-1 to bind to a dimer of its cofactor, SRF [28], and the D domain enables binding to MAP kinases of ERK, JNK, and p38 families. Meanwhile, the acidic domain (60–72) of MZF-1 on the entire sequence of (1–485) is where direct interaction with the heparin-binding domains (146–156) of Elk-1 on the entire sequence of (1–428) occurs via co-IP (preparing for publication). We therefore suggest that the cooperative interaction between MZF-1 and Elk-1 may be mediated through the acidic and heparin-binding domains, respectively, although the search for the binding sequence on Elk-1 is currently ongoing.

In this study, when we transfected MZF-1 or Elk-1 with a deleted DNA-binding domain to a cell, the expression of PKCα decreased, and the cells exhibited decreased potential proliferation/migration/invasion activity and tumorigenesis, thereby confirming the results of previous reports, in which gene knockdown of PKCα expression by antisense oligonucleotide or shRNA inhibits cell proliferation/migration/invasion [5–7]. We therefore propose the following model: more often than not, in tumor cells, the binding of MZF-1 and Elk-1 followed by clustering to the binding site of the PKCα promoter can stimulate PKCα expression. This activity can be inhibited by transfecting a DNA-binding domain truncated MZF-1 to the cell; the a DNA-binding domain truncated MZF-1 will bind to an endogenous Elk-1, or vice versa, thereby decreasing the number of successful endogenous Elk-1 and MZF-1 interactions and their binding to the PKCα promoter. Consequently, PKCα expression and cell growth and invasion decrease. Thus, the information pertaining to the influence of upstream genes of PKCα is expected to undergo further basic and clinical trials with alternative directions in the development of anti-cancer solutions.

## Supporting Information

S1 Fig Positive correlation of PKCα expression with transcription factors MZF-1 and Elk-1 in HCC cells. Transcription activity of PKCα promoter-driven luciferase and protein expression in five liver cancer cell lines by Luciferase assay and Western blotting (A). The luciferase expression of a 60bp PKCα promoter was observed in five HCC cells when transfected with 60bp PKCα promoter luciferase vector. Expression levels of PKCα, MZF-1 and Elk-1 proteins were estimated by Western blotting in five HCC cells. Huh-7 cells were transiently transfected with 1 μg of pGL3-60bp PKCα promoter luciferase reporter vectors and then the indicated amounts of empty pcDNA3 or MZF-1 expression vectors (B) and empty pcDNA3 or Elk-1 expression vectors (C). The bar below the two graphs displays Western blot of MZF-1 expression and Elk-1 expression. Five HCC cells were transiently co-transfected with 1 μg of 60 bp-PKCα promoter luciferase vector and 2.5 μg of MZF-1 or Elk-1 or MZF-1/Elk-1 expression vectors (D). Transcriptional activity is expressed as fold induction compared with the level obtained with each reporter vector in the absence of expression vectors. The experiments were repeated three times in triplicate. Data are means ± SE.
(TIF)

## References

[1] Martiny-Baron G1, Fabbro D. Classical PKC isoforms in cancer. Pharmacol Res. 2007;55: 477–486. 17548205 10.1016/j.phrs.2007.04.001

[2] Tam WL, Lu H, Buikhuisen J, Soh BS, Lim E, Reinhardt F, et al Protein kinase C α is a central signaling node and therapeutic target for breast cancer stem cells. Cancer Cell 2013;24: 347–364. 10.1016/j.ccr.2013.08.005 24029232 PMC4001722

[3] Hsu YH, Yao J, Chan LC, Wu TJ, Hsu JL, Fang YF, et al Definition of PKC-α, CDK6, and as therapeutic targets in triple-negative breast cancer. Cancer Res. 2014;74: 4822–4835. 10.1158/0008-5472.CAN-14-0584 24970481 PMC4154991

[4] Mackay HJ, Twelves CJ. (2007) Targeting the protein kinase C family: are we there yet? Nat Rev Cancer 2007;7: 554–562. 17585335 10.1038/nrc2168

[5] Hsieh YH, Wu TT, Tsai JH, Huang CY, Hsieh YS, Liu JY. PKCα expression regulated by Elk-1 and MZF-1 in human HCC cells. Biochem Biophys Res Commun. 2006;339: 217–225. 16297876 10.1016/j.bbrc.2005.11.015

[6] Hsieh YH, Wu TT, Huang CY, Hsieh YS, Liu JY. Suppression of tumorigenicity of human hepatocellular carcinoma cells by antisense oligonucleotide MZF-1. Chin J Physiol. 2007;50: 9–15. 17593797

[7] Ying TH, Hsieh YH, Hsieh YS, Liu JY. Antisense oligonucleotide Elk-1 suppresses the tumorigenicity of human hepatocellular carcinoma cells. Cell Biol Int. 2007;32: 210–216. 17950002 10.1016/j.cellbi.2007.08.027

[8] Wiren KM, Toombs AR, Zhang XW. Androgen inhibition of MAP kinase pathway and Elk- 1 activation in proliferating osteoblasts. J Mol Endocrinol. 2004;32: 209–226. 14766003 10.1677/jme.0.0320209

[9] Khurana A, Dey CS. Involvement of Elk-1 in L6E9 skeletal muscle differentiation. FEBS Lett. 2002;527: 119–124. 12220646 10.1016/s0014-5793(02)03192-7

[10] Vanhoutte P, Nissen JL, Brugg B, Gaspera BD, Besson MJ, Hipskind RA, et al Opposing roles of Elk-1 and its brain-specific isoform, short Elk-1, in nerve growth factor-induced PC12 differentiation. J Biol Chem. 2001;276: 5189–5196. 11050086 10.1074/jbc.M006678200

[11] Shao N, Chai Y, Cui JQ, Wang N, Aysola K, Reddy ES, et al Induction of apoptosis by Elk-1 and deltaElk-1 proteins. Oncogene 1998;17: 527–532. 9696047 10.1038/sj.onc.1201931

[12] Hromas R, Collins SJ, Hickstein D, Raskind W, Deaven LL, O'Hara P, et al A retinoic acid-responsive human zinc finger gene, MZF-1, preferentially expressed in myeloid cells. J Biol Chem. 1991;266: 14183–14187. 1860835

[13] Robertson KA, Hill DP, Kelley MR, Tritt R, Crum B, Van Epps S, et al The myeloid zinc finger gene (MZF-1) delays retinoic acid-induced apoptosis and differentiation in myeloid leukemia cells. Leukemia 1998;12: 690–698. 9593266 10.1038/sj.leu.2401005

[14] Hromas R, Morris J, Cornetta K, Berebitsky D, Davidson A, Sha M, et al Aberrant expression of the myeloid zinc finger gene, MZF-1, is oncogenic. Cancer Res. 1995;55: 3610–3614. 7627970

[15] Bavisotto L, Kaushansky K, Lin N, Hromas R. Antisense oligonucleotides from the stage-specific myeloid zinc finger gene MZF-1 inhibit granulopoiesis in vitro. J Exp Med. 1991;174: 1097–1101. 1719120 10.1084/jem.174.5.1097 PMC2119006

[16] Reid G, Hübner MR, Métivier R, Brand H, Denger S, Manu D, et al Cyclic, proteasome-mediated turnover of unliganded and liganded ERalpha on responsive promoters is an integral feature of estrogen signaling. Mol Cell. 200311: 695–707. 10.1016/s1097-2765(03)00090-x 12667452

[17] De Petro G, Tavian D, Copeta A, Portolani N, Giulini SM, Barlati S. Expression of urokinase-type plasminogen activator (u-PA), u-PA receptor, and tissue-type PA messenger RNAs in human hepatocellular carcinoma. Cancer Res. 1998;58: 2234–2239. 9605771

[18] Wu TT, Hsieh YH, Wu CC, Hsieh YS, Huang CY, Liu JY. Overexpression of protein kinase Cα mRNA in human hepatocellular carcinoma: a potential marker of disease prognosis. Clin Chim Acta. 2007;382: 54–58. 17459358 10.1016/j.cca.2007.03.018

[19] Hsieh YH, Wu TT, Huang CY, Hsieh YS, Hwang JM, Liu JY. p38 mitogen-activated protein kinase pathway is involved in protein kinase Calpha-regulated invasion in human hepatocellular carcinoma cells. Cancer Res. 2007;67: 4320–4327. 17483345 10.1158/0008-5472.CAN-06-2486

[20] Kannan-Thulasiraman P, Shapiro DJ. Modulators of inflammation use nuclear factor-kappa B and activator protein-1 sites to induce the caspase-1 and granzyme B inhibitor, proteinase inhibitor 9. J Biol Chem. 2002;277: 41230–41239. 12177049 10.1074/jbc.M200379200

[21] Bader GD, Hogue CW. Analyzing yeast protein-protein interaction data obtained from different sources. Nat Biotechnol. 2002;20: 991–997. 12355115 10.1038/nbt1002-991

[22] Clark JH, Haridasse V, Glazer RI. Modulation of the human protein kinase C alpha gene promoter by activator protein-2. Biochemistry 2002;41: 11847–11856. 12269829 10.1021/bi025600k

[23] Hromas R, Collins SJ, Hickstein D, Raskind W, Deaven LL, O'Hara P, et al A retinoic acid-responsive human zinc finger gene, MZF-1, preferentially expressed in myeloid cells. J Biol Chem. 1991;266: 14183–14187. 1860835

[24] Mitchell PJ, Tjian R. Transcriptional regulation in mammalian cells by sequence-specific DNA binding proteins. Science 1989;245: 371–378. 2667136 10.1126/science.2667136

[25] Ghosh S, Toth C, Peterlin BM, Seto E. Synergistic activation of transcription by the mutant and wild-type minimal transcriptional activation domain of VP16. J Biol Chem. 1996;271: 9911–9918. 8626627 10.1074/jbc.271.17.9911

[26] Gin P, Yin L, Davies BS, Weinstein MM, Ryan RO, Bensadoun A, et al The acidic domain of GPIHBP1 is important for the binding of lipoprotein lipase and chylomicrons. J Biol Chem. 2008;283: 29554–29562. 10.1074/jbc.M802579200 18713736 PMC2662032

[27] Fromm JR, Hileman RE, Caldwell EE, Weiler JM, Linhardt RJ. Pattern and spacing of basic amino acids in heparin binding sites. Arch Biochem Biophys. 1997;343: 92–100. 9210650 10.1006/abbi.1997.0147

[28] Besnard A, Galan-Rodriguez B, Vanhoutte P, Caboche J. Elk-1 a transcription factor with multiple facets in the brain. Front Neurosci. 2011;5: 35 10.3389/fnins.2011.00035 21441990 PMC3060702

