# Correction: MZF-1/Elk-1 Complex Binds to Protein Kinase Cα Promoter and Is Involved in Hepatocellular Carcinoma

**DOI:** 10.1371/journal.pone.0131036

**Published:** 2015-06-15

**Authors:** Chia-Herng Yue, Chih-Yang Huang, Jen-Hsiang Tsai, Chih-Wei Hsu, Yi-Hsien Hsieh, Ho Lin, Jer-Yuh Liu

There is an error in affiliation 9 for author Yi-Hsien Hsieh. Affiliation 9 should be: Department of Biochemistry, School of Medicine, Chung-Shan Medical University, Taichung, Taiwan.
