# Supplementary figures and images for: MZF-1/Elk-1 Complex Binds to Protein Kinase Cα Promoter and Is Involved in Hepatocellular Carcinoma
Source: PLoS One. 2015 May 26;10(5):e0127420. doi: 10.1371/journal.pone.0127420 (PMC4444300; doi:10.1371/journal.pone.0127420)

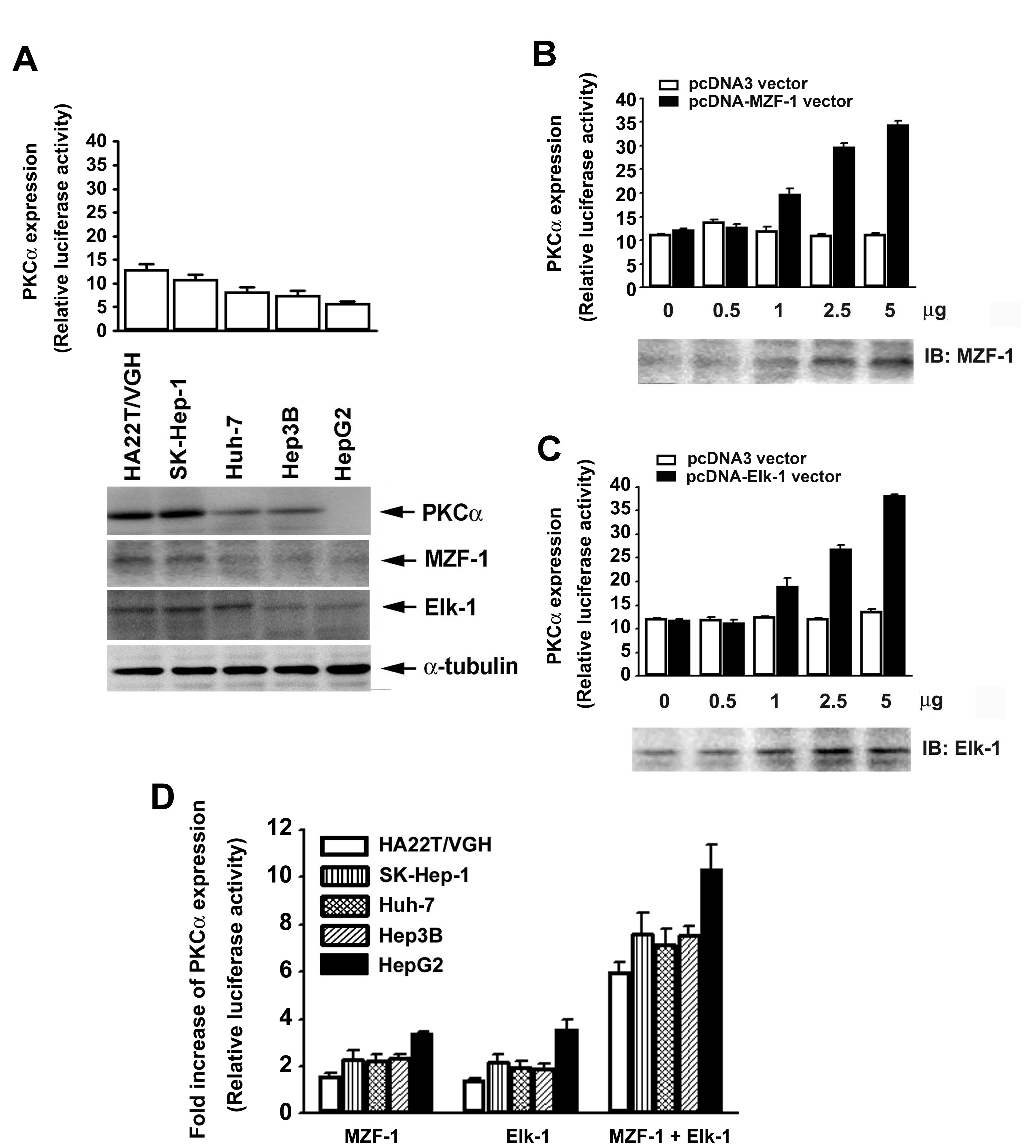

Supplement: S1 Fig — Transcription activity of PKCα promoter-driven luciferase and protein expression in five liver cancer cell lines by Luciferase assay and Western blotting (A). The luciferase expression of a 60bp PKCα promoter was observed in five HCC cells when transfected with 60bp PKCα promoter luciferase vector. Expression levels of PKCα, MZF-1 and Elk-1 proteins were estimated by Western blotting in five HCC cells. Huh-7 cells were transiently transfected with 1 μg of pGL3-60bp PKCα promoter luciferase reporter vectors and then the indicated amounts of empty pcDNA3 or MZF-1 expression vectors (B) and empty pcDNA3 or Elk-1 expression vectors (C). The bar below the two graphs displays Western blot of MZF-1 expression and Elk-1 expression. Five HCC cells were transiently co-transfected with 1 μg of 60 bp-PKCα promoter luciferase vector and 2.5 μg of MZF-1 or Elk-1 or MZF-1/Elk-1 expression vectors (D). Transcriptional activity is expressed as fold induction compared with the level obtained with each reporter vector in the absence of expression vectors. The experiments were repeated three times in triplicate. Data are means ± SE. (TIF) [file pone.0127420.s001.tif]
